# Caries risk profile of 12 year old school children in an Indian city using Cariogram

**DOI:** 10.4317/medoral.17880

**Published:** 2012-08-28

**Authors:** Mamata Hebbal, Anil Ankola, Sharada Metgud

**Affiliations:** 1 Reader and Ph.D scholar, Dept of Public Health Dentistry; 2 Professor and Head, Dept of Public Health Dentistry; 3 Professor and Former Head, Dept. of Microbiology

## Abstract

Objectives: The present study was conducted with an aim to assess the caries profile of 12 year old Indian children using Cariogram. Study design: Hundred children were interviewed to record any illness, oral hygiene practices and fluoride exposure after obtaining a three day diet diary. Examination was done to record plaque and dental caries status. Stimulated saliva was collected and salivary flow rate, salivary buffering capacity, Streptococcus mutans and Lactobacillus were assessed. The information obtained was scored and Cariogram was created. Differences between mean decayed, missing and filled teeth ( DMFT) and Cariogram risk groups were assessed using ANOVA. Spearman Correlation coefficients were used to explore correlation among Cariogram scores and individual variables. Results: It was found that 21, 45, 21 and 13 children had 0-20%, 21-40%, 41-60% and 61-100% chance of avoiding caries respectively in future. Significant correlation was observed between cariogram score and DMFT, diet content, diet frequency, plaque scores, Streptococcus mutans counts and fluoride programme. Conclusions: Cariogram model can identify the caries-related factors that could be the reasons for the estimated future caries risk, and therefore help the dentist to plan appropriate preventive measures.

** Key words:**Cariogram, caries risk assessment, risk factors, children, India

## Introduction

Dental caries is defined as a progressive, irreversible microbial disease of multifactorial nature affecting the calcified tissues of the teeth characterized by demineralization of the inorganic portion and destruction of the organic portion of the tooth. It is a disease of civilization. Almost all people are affected by dental caries, only the severity differs. There is interplay of three principal factors, the host, the micro flora and the substrate or diet in the occurrence of dental caries. In addition, the fourth factor time must be considered in any discussion regarding etiology of caries. For caries to occur conditions related to each of these factors must be favourable. Dental caries can be prevented by applying suitable measures, hence it is very important to identify those individuals who are most likely to develop dental caries through caries risk assessment, and provide them the required preventive measures to interrupt the disease process. The multifactorial etiology of dental caries points to a risk assessment model that would include the different factors or parameters that accompany the development of new carious lesions. Cariogram is one such model which assesses and illustrates a caries risk profile for an individual graphically, simultaneously taking into account the interaction of different caries causing factors/parameters of the patient (1,2).

Cariogram was presented in 1996 by Bratthall (1) for illustrating the interactions of caries related factors. The model makes it possible to single out individual risk or resistance factors. A special interactive version for the estimation of caries risk has been developed. The original Cariogram was a circle divided into three sectors, each representing factors strongly influencing carious activity: diet, bacteria, and susceptibility. The development of the model was based on the need to explain why, in certain individuals, caries activity could be low in spite of other influencing factors like high sucrose intake, poor oral hygiene, high Streptococci load, or nonuse of fluorides.

Based on the Cariogram concept, an interactive version for caries risk estimation was developed Bratthall et al., in 1997 (1). There are a few fundamental differences between this program and the original version. First, the risk for future caries activity varies on a scale from 0-100%, but it cannot be more than 100%. Thus, the sectors cannot overlap each other. Second, a further sector, circumstance, was included. This sector includes factors such as caries experience and systemic diseases factors to consider when the risk is calculated, in spite of the fact that these factors themselves do not participate directly in the development of the lesion. Since very few studies have been conducted on Cariogram and only one Indian study has been published (3) the present study was conducted with an aim to evaluate the caries profile of Indian children using Cariogram.

## Material and Methods

The present study was conducted among 12 years old school children of Belgaum city, which is located in the southern part of India. This is a part of the longitudinal study which is being conducted to assess the effect of preventive measures on caries risk. There are a total of 285 schools in Belgaum city. A total of four schools were randomly selected for the study. Permission to conduct the study was obtained from Deputy Director of Public Instructions (DDPI) and school authorities. Ethical clearance was also obtained from Institutional Review Board (IRB). Informed consent and assent was obtained from parents and children respectively.

The study was conducted for a period of three months from July to September 2008. It followed the Cariogram model with few modifications to be applicable for field study and Indian scenario. It consisted of five steps 

1. Questionnaire: The questionnaire was given to those children whose parents gave informed consent. The questionnaire mainly pertained to personal details along with three day diary (including Sunday) to be filled by the children with the assistance of parents. Children were shown an example of recording diet diary. They were instructed to complete and return the questionnaire on fourth day.

2. Interview: The questionnaires were collected and children were individually interviewed to record any illness, oral hygiene practices and fluoride exposure. As children may not reveal easily about any illness and may not know whether the toothpaste was fluoridated interview was thought to be an appropriate method. This also helped to ensure that the questionnaire was duly filled.

3. Clinical examination: Children were examined for plaque and dental caries. Silness and Loe plaque index (4) was used to assess the amount of plaque. Dental caries was assessed using WHO Dentition Status and Treatment Needs in which caries experience was calculated by adding decayed, missing and filled permanent teeth (5). A single trained and calibrated examiner recorded both the indices. The intra examiner reliability was found to be 0.78 and 0.86 respectively for both the indices respectively.

4. Saliva collection: Simplified techniques of salivary assessments were used to make them cost effective and applicable for the field study. Children were asked to chew a modeling wax made into a form of pellet (0.5 x0.5 centimeters) for 3 minutes to obtain stimulated saliva.

i. Salivary flow rate: Saliva from oral cavity was sucked using a sterile disposable syringe and amount of saliva secreted per minute was calculated.

ii. Salivary buffering capacity: 0.5ml of saliva was added to 1.5ml of 0.005 molarity of hydrochloric acid (HCL). Buffering capacity of saliva was determined by assessing the change in pH using commercially available Indikrom paper, which have a predetermined pH range and categorized accordingly.

iii. Microbial assessment: By means of a sterile disposable syringe 0.5 ml aliquot of saliva collected directly from the oral cavity was injected in a previously labeled sterile bottle containing 2ml of transport medium. The samples were processed on the same day in the Department of Microbiology, Jawaharlal Nehru Medical College.

Laboratory procedure: The samples were vortexed to uniformly mix the saliva and the media using a cyclomixer. Using an inoculation loop (standard loop with 4mm inner diameter) 10 ml of the vortexed sample was streaked on Mitis salivarius agar selective for Streptococcus mutans and on Rogosa SL agar for Lactobacillus. The Mitis salivarius agar plates were incubated in an anaerobic jar for 48 hours at 37°C in an incubator and similar procedure were followed for Rogosa SL agar plates, which were incubated for 96 hours.

**Figure 1:**
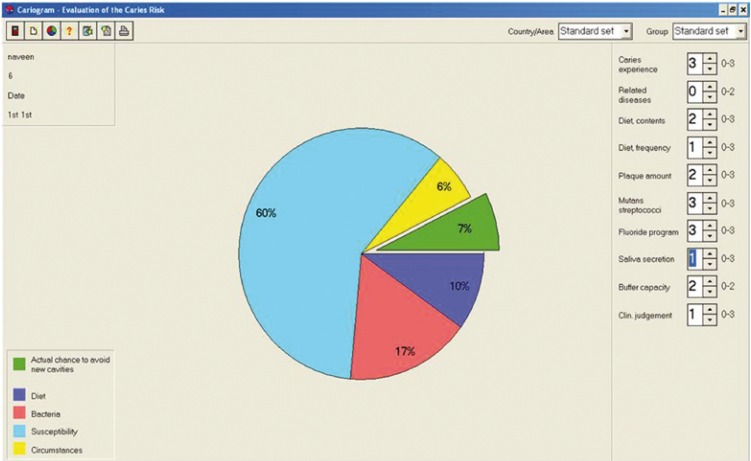
Example of Cariogram from the present study illustrating a very high risk for caries (with 7% chance of avoiding caries).

5. Creation of Cariogram: When all the information was available they were scored according to the predetermined scale as 0-2 or 3 ( Table 1). The scores were entered into the cariogram computer programme to calculate the ‘caries risk’ and conversely ‘chance of avoidance of caries’ for each child. An example of cariogram of a child is shown in figure 1.

**Table 1:**
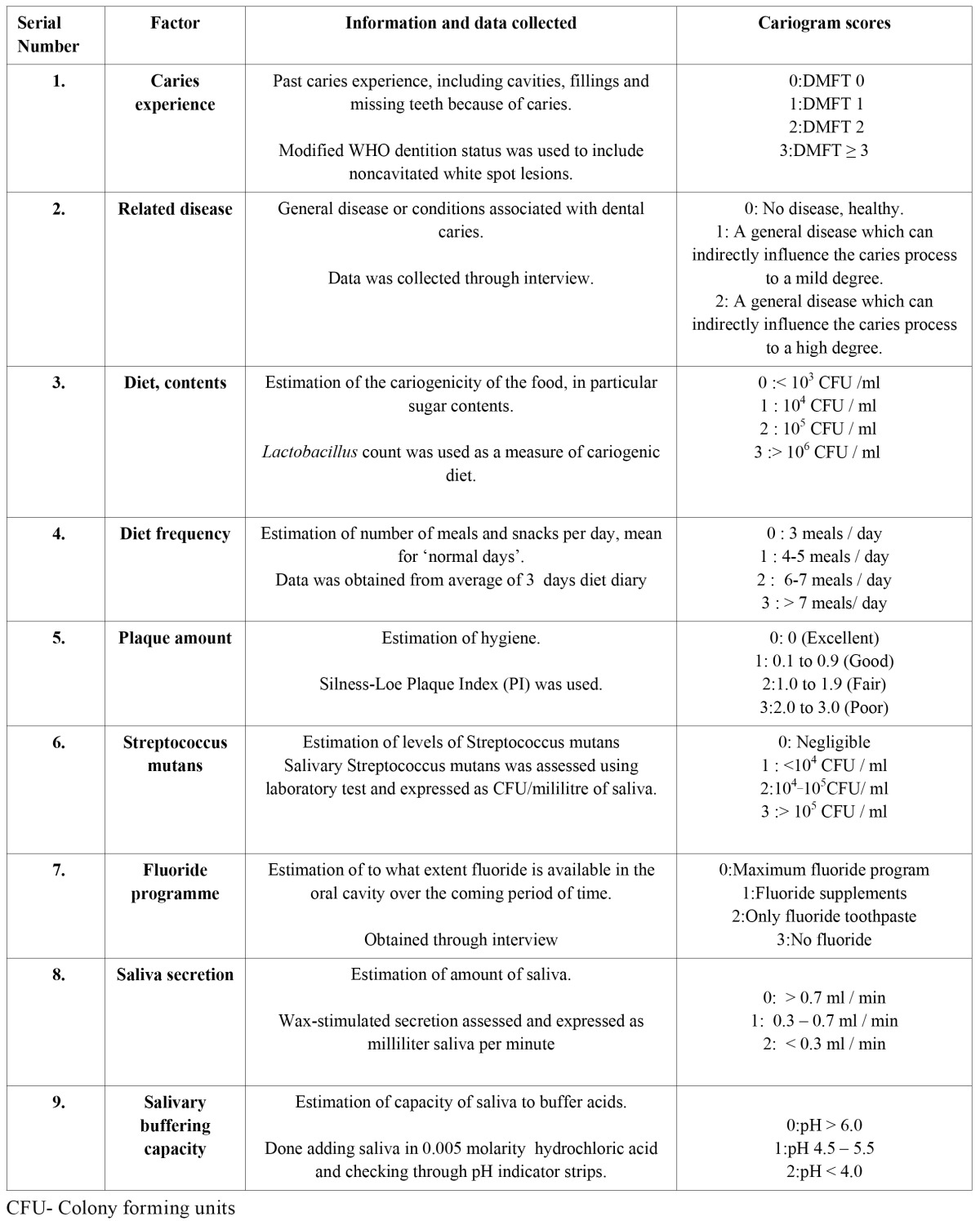
Caries related factors used for the Cariogram.

Statistical methods: All the data were analysed using SPSS statistical package (version 17.0 SPSS Inc. Chicago III, USA). Descriptive statistics including the means and standard deviations of all caries related factors were calculated for all four caries related groups. Chi square test was used to find differences between caries related factors and Cariogram group. Differences between mean decayed, missing and filled teeth (DMFT) and Cariogram groups was assessed using ANOVA. Spearman Correlation coefficients were used to explore associations among Cariogram scores and individual variables. P-value <0.05 was considered statistically significant.

## Results

The present study was conducted among 100, 12 year old children comprising 53 males and 47 females.  Table 2 represents distribution of study subjects according to percentage chance of avoiding caries and caries related factors. When children were grouped according to chance of avoiding caries it was found that 21, 45, 21 and 13 children had 0-20%, 21-40%, 41-60% and 61-100% chance of avoiding caries respectively in future. Chi square test revealed that there was statistically significant association between past caries experience, diet contents, plaque amount, Streptococcus mutans and fluoride programme and chance of avoiding caries were associated which was statistically significant whereas no statistical significance was found for diet frequency, saliva secretion and buffering capacity(p=0.16,p=0.34,p=0.82 respectively).

**Table 2:**
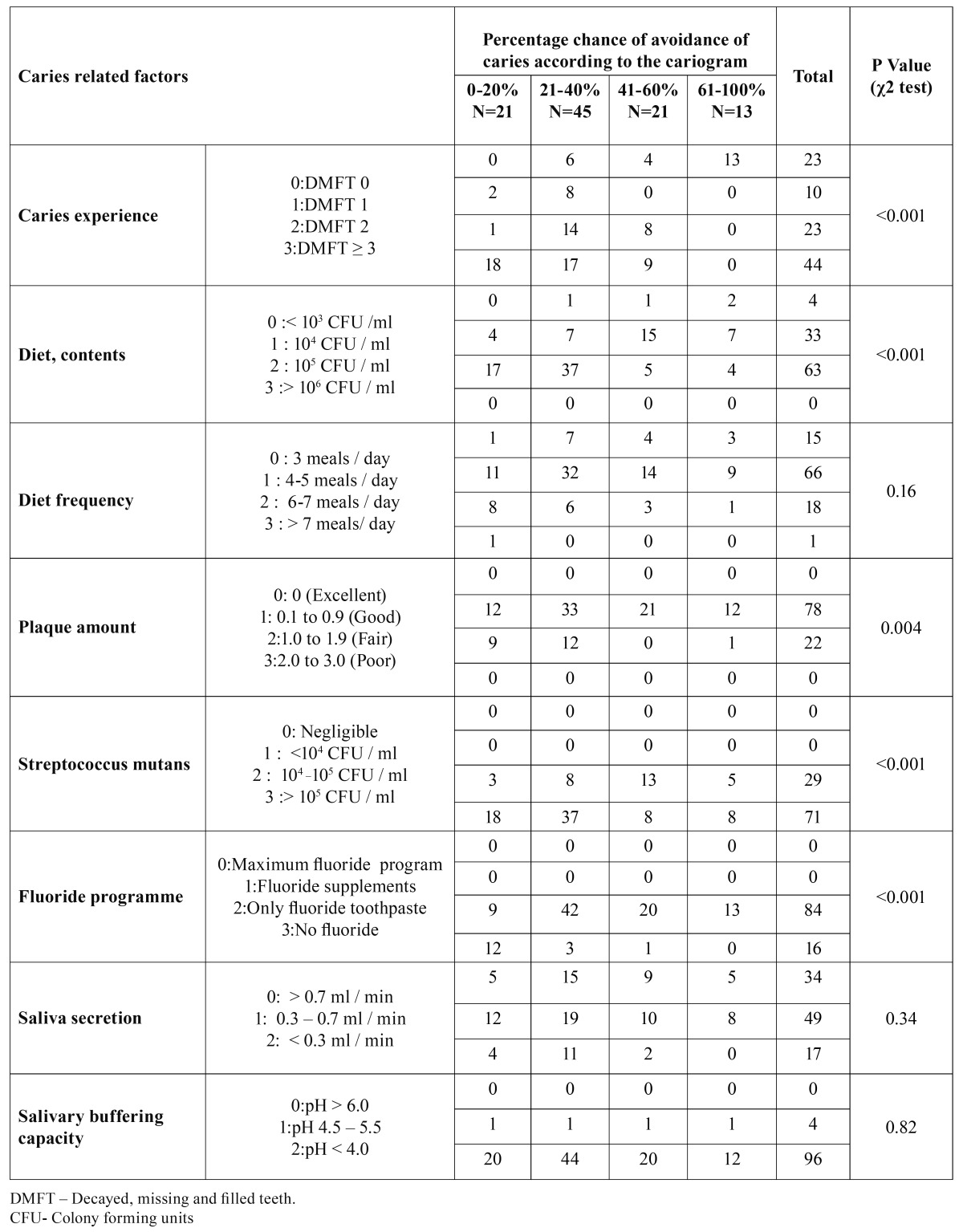
Distribution of study subjects according to caries related factors of Cariogram and percentage chance of avoiding caries.

The mean DMFT was 2.69 (±2.34) and the mean DMFT among males and females was 2.62 (±2.22) and 2.76(±2.49) respectively. Student’s t test revealed no statistically significant difference among both the groups (p=0.76). The mean DMFT in the high risk group was 4.47 (±2.08) and low risk group had no dental caries. A reduction in mean DMFT was found in accordance with a rise in the likelihood of new caries being avoided in the near future (from highest to the lowest risk group) which was statistically significant(p<0.001) as shown in  Table 3. Significant correlation was observed between Cariogram score and DMFT, diet content, diet frequency, plaque scores, Streptococcus mutans counts and fluoride programme ( Table 4).

**Table 3:**
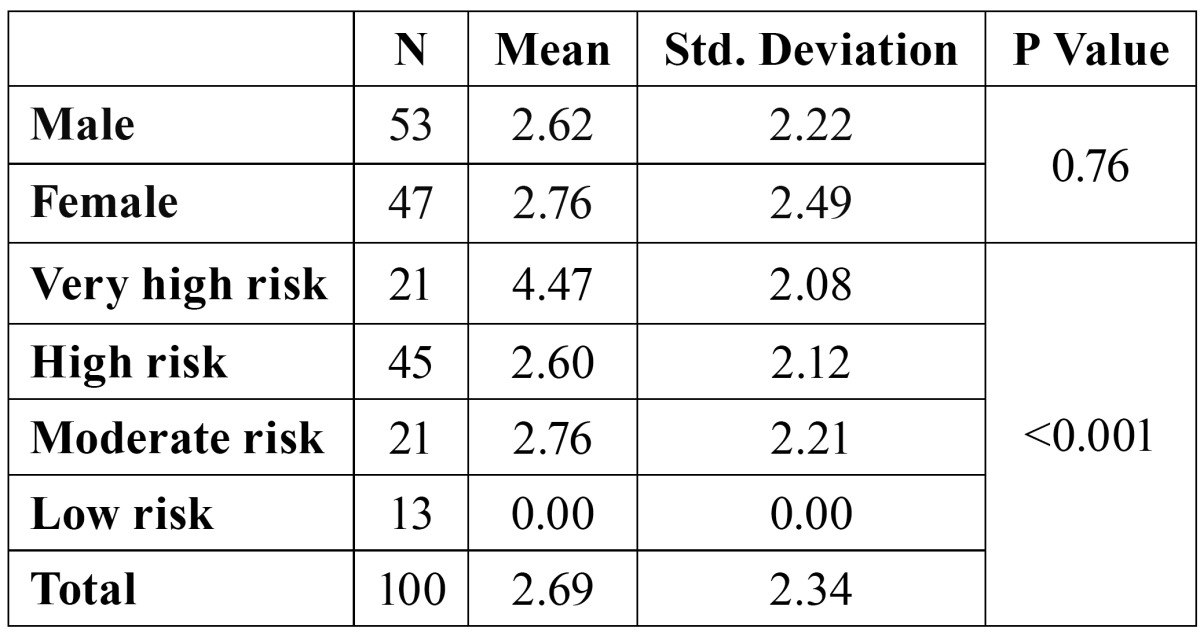
Distribution of study subjects according to gender, categorization with cariogram and mean decayed missing and filled teeth (DMFT).

**Table 4:**
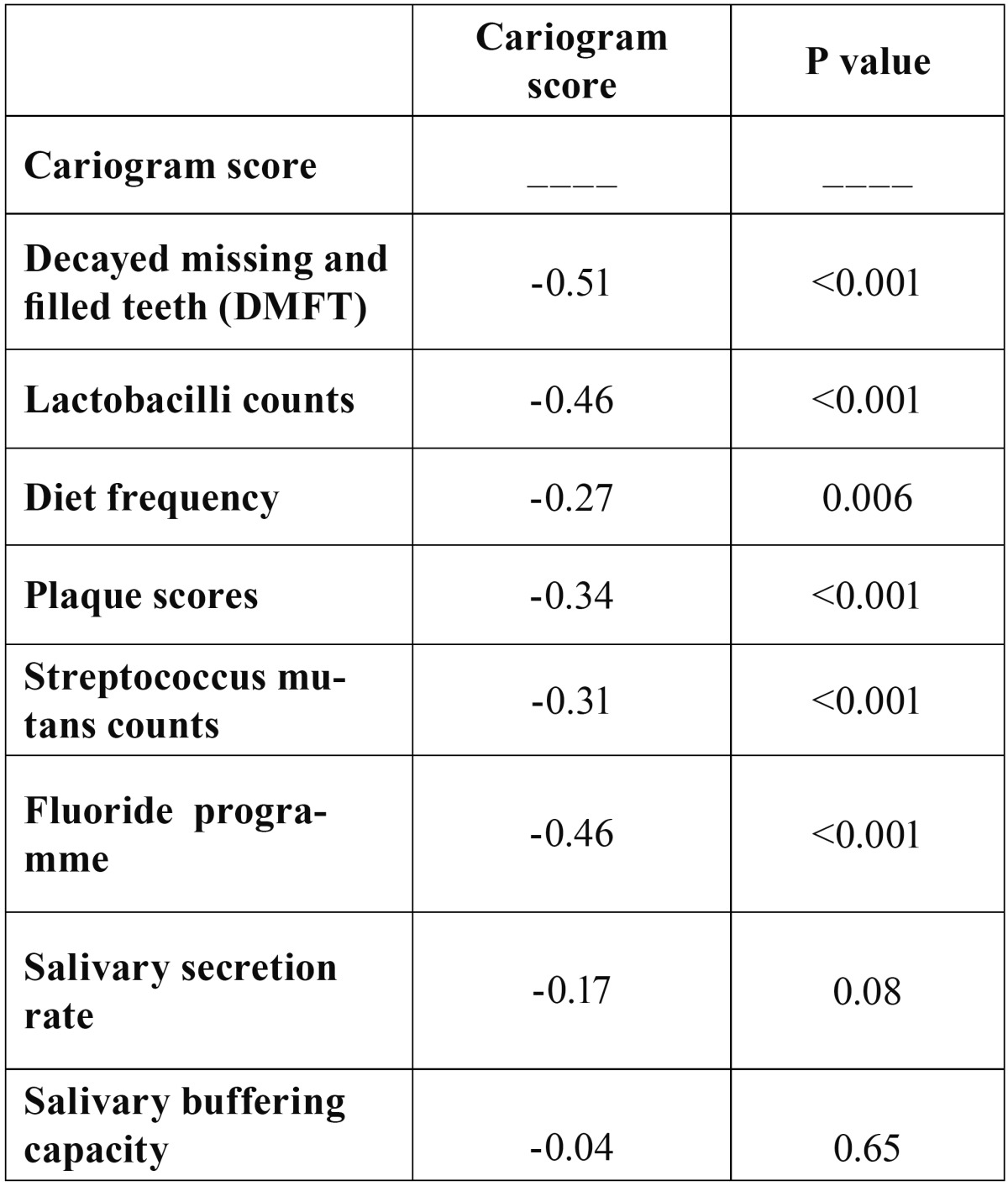
Correlation between cariogram score and caries related factors.

## Discussion

The present study was conducted among 12 year old school children to evaluate the caries profile using Cariogram. The age group of 12 year old was chosen as this is a WHO global monitoring age for dental caries, and only children with permanent dentition were selected in order to avoid discrepancies between mixed and permanent dentition with regard to microbial counts as stated by Schlagenhauf et al.,(6). Children in the present study had relatively low dental caries expressed as mean DMFT although the prevalence was 77 %. This in accordance with study conducted by Mascarenhas et al.,(7) who found that 22% of children were free of dental caries and mean DMFT and DMFS were 2.78 and 4.20 respectively.

The present study used Cariogram, which is considered one of the most reliable models as reported by many authors (2,8-11), for predicting caries risk in an individual because it is an objective, quantitative method that uses a computer program to calculate the data, with results that can be printed out and saved. Another advantage is that it makes a series of recommendations for preventive action according to the caries risk. The pie chart presentation with its different sectors makes it easier for patients to understand and can be effectively used to motivate the patient. They can be used to improve the comprehension of the factors that are having or could have a negative effect on their oral health (8). Cariogram has been investigated and validated for use in both children and elderly individuals (2,9). Nevertheless, Cariogram has been used in several cross-sectional studies (8,10,11). Since a single study has been published among Indian population with a small sample size (3) the present study was designed to classify the population according to the risk of developing caries in the future using Cariogram and to identify the caries-related factors contributing to the risk.

In the present study none of the children had 80-100% chance of avoidance of caries hence low and very groups were combined together and made as 60-100% chance of avoiding of caries and finally there were only four groups instead of five groups. It is not uncommon in India that people do not avail dental treatment regularly. Utilization of preventive dental services is negligible as they give very low priority for oral health and visit dentist only when in pain (12,13). This could explain the reason for absence of children in very low risk group which is in contrast to the study conducted by Hänsel Petersson et al., (2) where 40% of children belonged to very low risk (2).Similar results were obtained in a study conducted by Campus et al., (14) .Of the various car-ies-related factors included in the Cariogram model, three obtained high Cariogram scores (i.e., 2-3) in the majority of patients. These factors were past caries experience, Streptococci mutans, and use of fluoride. These factors could explain both the high caries status of the individuals and the probability of having a high risk of developing caries lesions in the future. Similar results were obtained in a study conducted by Sonbul et al., where other factors like lactobacilli, plaque amount had high Cariogram scores (15).

A reduction in mean DMFT found in accord with a rise in the likelihood of new caries being avoided in the near future (from highest to the lowest risk group) was comparable to the results of Tayanin et al., (10) and Campus et al., (14), et al where similar pattern was observed for dmfs. Past caries experience has often been considered as caries risk factor. Many authors over the years have strongly advocated that this is the best predictor, better than other prognostic variables (16-18). It is imperative to realize that the past caries is the effect and not the cause of caries disease. If effective interventions are introduced and risk factors eliminated, then past caries experience loses its predictive role. The fact that this variable is still so powerful in so many studies rather reflects that the caries normally is not controlled adequately or that routine preventive measures are not effective enough (19).

In the present study none of children were suffering from any systemic disease or condition which may directly or indirectly affect the caries process which is due to the fact that the study was conducted among young children. Hence the Cariogram considered zero score for related disease for all the subjects and no correlation was found. These results are in accordance with the study conducted by Ruiz-Miravet et al., (8).

Exposure to fluoride is one of the most important protective factors when evaluating caries risk and is the cause of the considerable fall in caries levels in Western countries (20). None of the children in this study used fluoride supplements and the only source of fluoride was fluoridated tooth pastes, use of which was confirmed by asking the brand name. All the children consumed water from municipal water supply and fluoride content of which is very minimal. Eighty four percent of children used fluoridated toothpastes though they did not really know its purpose and existence in tooth pastes. This reflects less motivation and awareness rendering them more likely to run a high risk of caries (15). Similar results were obtained in study conducted by Tayanin et al., (10).

Few modifications were made in salivary collection and assessment. Stimulated saliva was collected for microbial assessment using modeling wax made in the form of pellets of standard size. In the pilot study various materials like paraffin wax, chewing gum base and orthodontic bands were also used to stimulate saliva, and children found modeling wax to be more attractive which could be due to the colour. Microbial assessment showed no difference in the counts irrespective of the material used for stimulation. Precaution was taken to avoid contamination by direct collection of saliva from the oral cavity through syringe rather than spitting in the cup. The saliva was immediately added to thioglycolate transport medium containing vial and processed on the same day rather than using Dentocult SM, Dentocult LB or Dentobuff strip as this procedure is more economical and reliable results can be obtained. Significant results were obtained for Streptococcus mutans, lactobacilli but not for salivary flow rate and buffering capacity. Some studies suggest that children with high counts of Streptococcus mutans or Lactobacilli or low salivary buffer capacity often show higher DMFT values (21,22). Oral hygiene on the other hand, often shows only a weak correlation with dental caries(23). In contrast, few studies have reported that the efficacy of salivary Streptococcus mutans colonies as predictors of future caries is 50% in the general population and even smaller in populations with low caries rates (24), as also occurs with salivary buffer capacity (25).

Spearman correlation coefficient was used to find the correlation between the different variables and the risk obtained for each sector. Significant correlation was obtained for all the variables except related diseases, salivary secretion rate and buffering capacity. Similar results were obtained in a studies conducted by Ruiz-Miravet et al., (8) and Campus et al., (14) who obtained no significant correlation only for related diseases.

The clinical judgement variable removes the objectivity that the Cariogram should show when assessing a patient’s caries risk, as it makes it possible to alter the risk result by applying a subjective opinion. Consequently, in the present research this variable was set throughout at 1, which means that the examiner does not have any reason to change the program’s evaluation as the caries situation, including social factors, gives a similar impression to the Cariogram program. With 1 as the value the percentages given by the Cariogram were not altered, so an objective result was obtained.

To conclude, variables like caries experience, Lactobacillus counts, Streptococcus mutans, diet frequency, fluoride programme and plaque amount included in the Cariogram presented significant correlation with the caries risk determined by this program. The Cariogram model can identify the caries-related factors that could be the reasons for the estimated future caries risk, and therefore help the dentist to plan appropriate preventive and treatment measures in daily clinical practice.
